# Prevalence and Persistence of Multidrug-Resistant *Yersinia enterocolitica* 4/O:3 in Tonsils of Slaughter Pigs from Different Housing Systems in Croatia

**DOI:** 10.3390/foods11101459

**Published:** 2022-05-18

**Authors:** Nevijo Zdolec, Marta Kiš, Dean Jankuloski, Katerina Blagoevska, Snježana Kazazić, Marina Pavlak, Bojan Blagojević, Dragan Antić, Maria Fredriksson-Ahomaa, Valerij Pažin

**Affiliations:** 1 Faculty of Veterinary Medicine, University of Zagreb, 10000 Zagreb, Croatia; mkis@vef.hr (M.K.); mpavlak@vef.hr (M.P.); vpazin@vef.hr (V.P.); 2 Faculty of Veterinary Medicine, Food Institute, 1000 Skopje, North Macedonia; djankuloski@fvm.ukim.edu.mk (D.J.); kblagoevska@fvm.ukim.edu.mk (K.B.); 3 Ruder Boskovic Institute, 10000 Zagreb, Croatia; snjezana.kazazic@irb.hr; 4 Faculty of Agriculture, Department of Veterinary Medicine, University of Novi Sad, 21000 Novi Sad, Serbia; blagojevic.bojan@yahoo.com; 5 Faculty of Health and Life Sciences, Institute of Infection, Veterinary and Ecological Sciences, University of Liverpool, Leahurst, Neston CH64 7TE, UK; dragan.antic@liverpool.ac.uk; 6 Faculty of Veterinary Medicine, University of Helsinki, 00014 Helsinki, Finland; maria.fredriksson-ahomaa@helsinki.fi

**Keywords:** *Yersinia enterocolitica* 4/O:3, pigs, slaughter, farm, antimicrobial resistance

## Abstract

*Yersinia enterocolitica* is one of the priority biological hazards in pork inspection. Persistence of the pathogen, including strains resistant to antimicrobials, should be evaluated in pigs from different housing systems for risk ranking of farms. In this 2019 study, tonsils were collected from 234 pigs, of which 69 (29.5%) were fattened on 3 big integrated farms, 130 (55.5%) on 10 medium-sized farms, and 35 (15%) on 13 small family farms. In addition, 92 pork cuts and minced meat samples from the same farms were tested for the presence of *Y. enterocolitica* using the culture method. Phenotypic and genetic characteristics of the isolates were compared with previously collected isolates from 2014. The overall prevalence of *Y. enterocolitica* in pig tonsils was 43% [95% CI 36.7–49.7]. In pigs from big integrated, medium-sized, and small family farms, the prevalence was 29%, 52%, and 40%, respectively. All retail samples of portioned and minced pork tested negative for pathogenic *Y. enterocolitica*, likely due to high hygienic standards in slaughterhouses/cutting meat or low sensitivity of culture methods in these matrices. The highest recovery rate of the pathogen from tonsils was found when alkali-treated PSB and CIN agar were combined. The biosecurity category of integrated and medium farms did not affect the differences in prevalence of *Y. enterocolitica* (*p* > 0.05), in contrast to family farms. Pathogenic *ail*-positive *Y. enterocolitica* biotype 4 serotype O:3 persisted in the tonsils of pigs regardless of the type of farm, slaughterhouse, and year of isolation 2014 and 2019. PFGE typing revealed the high genetic concordance (80.6 to 100%) of all the *Y. enterocolitica* 4/O:3 isolates. A statistically significant higher prevalence of multidrug-resistant *Y. enterocolitica* 4/O:3 isolates was detected in the tonsils of pigs from big integrated farms compared to the other farm types (*p* < 0.05), with predominant and increasing resistance to nalidixic acid, chloramphenicol, and streptomycin. This study demonstrated multidrug resistance of the pathogen in pigs likely due to more antimicrobial pressure on big farms, with intriguing resistance to some clinically relevant antimicrobials used in the treatment of yersiniosis in humans.

## 1. Introduction

Yersiniosis is one of the leading zoonoses in Europe, caused by pathogenic *Yersinia enterocolitica* bioserotypes and mainly transmitted through contaminated food. The pooled global prevalence of *Y. enterocolitica* in cases of human gastroenteritis has been recently estimated to be 1.97% [95% CI 1.32–2.74%], dominated by serotype O:3 [1]. According to the latest data from EFSA and ECDC Zoonoses Report, reporting data for 2020, there were 5668 human cases of this disease reported in Europe, with very limited surveillance data in the meat production chain [2]. In addition, six European countries reported only 0.2% of pigs (out of 2351 tested) positive for *Y. enterocolitica*, but these data were most likely related to fecal testing on farms. A total of 12.5% of pork sold at retail and 4.7% of samples (carcass swabs, pork) from cutting plants and slaughterhouses were *Y. enterocolitica* positive [2].

The main carriers of pathogenic *Y. enterocolitica* are pigs, with their tonsils being the main predilection site [3]. The reported prevalence of the pathogen in pigs varies widely among the numerous studies, which is to be expected considering the many risk factors involved from farm to slaughterhouse. Virtanen et al. [4] reported that factors contributing to fecal shedding of *Y. enterocolitica* include carriage of pathogen on the tonsils, purchase of feed from different suppliers, fasting of pigs prior to transport to slaughter, and snout contacts. Furthermore, Vilar et al. [5] claimed that the prevalence of *Y. enterocolitica* in pigs can only be reduced by supplying water of municipal origin and applying the “all-in-all-out” method, while risk factors contributing to increase were a lack of bedding and sourcing piglets from multiple farms. Existing pig farming systems differ significantly in terms of biosecurity levels and could, therefore, pose differing animal health risks. For example, important aspects include the transmission of *Y. enterocolitica* at the interface between livestock and wildlife and the role that wild and peridomestic rodents play as a source of this zoonotic pathogen for pigs [6]. Regarding the possibility of meat contamination during slaughter, Vilar et al. [7] indicated that risk factors include the presence of *Y. enterocolitica* in the intestines (OR: 35.6, 95% CI 2.8–8285), tonsils (OR: 38.4, 95% CI 5.0–854), and offal (OR: 16.6, 95% CI 1.9–1111). Furthermore, differences between slaughterhouses, where different hygiene practices are applied during slaughter and dressing, could increase cross-contamination from tonsils to carcasses [8]. In addition to farm- and slaughterhouse-related risk factors, differences in reported prevalences among studies could also be due to pathogen isolation methods. Therefore, traditional isolation methods are supplemented with more sensitive and rapid techniques such as polymerase chain reaction (PCR) screening. Additionally, matrix assisted laser desorption ionization-time of flight mass spectrometry (MALDI-TOF MS), PCR, pulsed-field gel electrophoresis (PFGE), multiple locus variable number of tandem repeats analysis (MLVA), and sequencing have been widely used for identification and characterization of *Yersinia* isolates [9].

*Y. enterocolitica* biotypes and serotypes associated with pathogenicity occur in both pigs and infected humans, with bioserotype 4/O:3 being the most common in continental Europe [10]. Consumption of raw and inadequately heat-treated pork and untreated water are considered the main risk factors for human infection [10]. Although pork is considered the main source of human infection; many studies have shown that pathogenic *Y. enterocolitica* is rarely found in portioned pork on the market, except for carcass parts and organs that are more likely to be contaminated at slaughter (cheeks, head, tongue, throat) [11]. However, the pathways of contamination and persistence of pathogenic strains have been confirmed over years in the pork production chain, linking the farm and the pork produced [12,13]. In recent years, research on antimicrobial resistance in foodborne pathogens has intensified to reduce the spread of resistance in the food chain. *Y. enterocolitica* is generally sensitive to clinically relevant antibiotics, and similar resistance profiles persist over time, which is explained by the genetic stability of the bacterium [14]. However, recent reports warn of foodborne yersiniosis outbreaks associated with multidrug-resistant *Y. enterocolitica* 4/O:3, which possess resistance genes of major public health concern that are acquired by horizontal transfer [15].

Therefore, the aim of this study was to determine the prevalence of (multidrug-resistant) *Y. enterocolitica* in the tonsils of slaughtered fattening pigs raised in different housing systems: big integrated farms, cooperative farms (medium-sized farms), and small family farms in Croatia. In addition, the presence of the pathogen on the market was evaluated in portioned pork and minced meat that originated from the investigated farms. The study also aimed to determine the persistence of the pathogen in the pork production chain by comparing the phenotypic and genetic characteristics of *Y. enterocolitica* with previously collected isolates in Croatia [16].

## 2. Materials and Methods

### 2.1. Farms and Slaughterhouses Included in the Study

All pigs included in this study originated from fattening farms, and were slaughtered in the same slaughterhouses as in previous survey from 2014. Three types of pig farms were included in the study: big integrated farms (>10,000 pigs), medium-sized farms (300–10,000 pigs), and small family farms (<300 pigs). The biosecurity category of investigated farms was obtained from the national database of registered farms; category 3 contains the farms with the highest biosecurity level, category 2 indicates that some biosecurity improvements are needed, and category 1 contains the farms with a low biosecurity level. A survey of the farms regarding their biosecurity levels was not conducted as a part of this study.

The big integrated farms involved (*n* = 3) used a vertical management system, their own piglets from separated breeding farms, their own produced crops and feed, and high biosecurity standards. The number of fattening pigs (per year) in these farms ranged from 11,000 to 31,000. Medium-sized farms (*n* = 10) purchased piglets from different local farms and import. The level of biosecurity in the medium-sized farms was medium to high. The number of fattening pigs on the investigated medium-sized farms ranged from 600 to 3000. Small family farms (*n* = 13) had their own sows and piglets that were fattened for slaughter. These farms had lower biosecurity conditions. The number of pigs on these farms ranged from 6 to 300.

Selected characteristics of the slaughterhouses involved in the study are shown in Table 1. Slaughterhouses were categorized as low, medium, or high risk based on the following parameters: slaughterhouse capacity and size of meat distribution area (factor of 0.30), past non-compliance in terms of infrastructure, equipment and hygiene (factor of 0.40), and the degree of implementation of HACCP principles and animal welfare rules (factor of 0.30) [17].

### 2.2. Sampling of Tonsils and Retail Meat 

Tonsils from 234 fattening pigs were collected by simple random sampling after pluck set removal in four slaughterhouses during 12 sampling sessions (slaughterhouse 1—pigs from three big integrated farms (*n* = 69); slaughterhouses 2, 3, 4—pigs from 10 medium-sized farms (*n* = 130), and slaughterhouse 3—pigs from 13 family farms (*n* = 35); Table S1—Supplementary Materials). 

A total of 92 samples of retail pork cuts (neck, thigh, loin, shoulder, bacon) and minced pork, originating from the investigated farms, were tested. These samples were obtained from local markets/supermarkets owned by the same companies that owned the slaughterhouses. In addition, 36 samples were obtained from other local producers and from import. Tonsil and meat samples were transported refrigerated to the laboratory and analyzed within 30 min of arrival. The maximum time from sample collection to analysis was 3 h.

### 2.3. Microbiological Analyses of Tonsils and (Minced) Pork

Ten grams of each tonsil (*n* = 234) and meat sample (*n* = 128) were homogenized in 90 mL of enrichment broth (peptone, sorbitol, and bile salts, PSB, Sigma Aldrich, St. Louis, MO, USA), of which 10 mL was transferred to 90 mL of selective enrichment broth (Irgasan^TM^ Ticarcillin and Potassium chlorate, ITC, Sigma Aldrich, St. Louis, MO, USA). Subsequently, both solutions were incubated at 25 ± 1 °C for 44 ± 4 h followed by streaking on Cefsulodin, Irgasan^TM^, and Novobiocin agar (CIN, Merck, Darmstadt, Germany) and CHROMagar^TM^ *Y. enterocolitica* (Paris, France). Broths cultures were then treated with alkaline solution (0.5% KOH) for 20 s, and streaked again on the same selective agars, incubated for 24 ± 2 h at 30 ± 1 °C [16]. Characteristic colonies on CIN agar (small, round, smooth, with dark red center and transparent edge—“bull’s eye”) were retained and subcultured for further identification and characterization. Colonies that were CHROMagar^TM^ purple (presumptive pathogenic) were also retained and subcultured. The alkali treatment of broth cultures was considered a risk factor for unsuccessful isolation of *Y. enterocolitica* on selective media. The odds ratio of the events (isolation and failed isolation of *Y. enterocolitica*) was calculated in relation to the prevalence detected after alkali treatment.

### 2.4. Assessment of Y. enterocolitica Persistence

Selected isolates of *Y. enterocolitica* obtained from this study (*n* = 84) were compared for phenotypic and genetic characteristics with selected isolates (*n* = 49) from a previous survey conducted in the same slaughterhouses and in pigs originated from comparable housing systems [16]. A total of 84 isolates were selected from 101 positive tonsils in this study for further characterization, representing all positive batches and farms. All isolates from the tonsils of pigs kept on small family farms were retained for further analysis (1–3 positive tonsils per farm). For medium and big farms, a maximum of seven isolates from one farm were retained (2 to 12 positives per farm).

#### 2.4.1. Identification of Isolates by MALDI-TOF MS and Real Time PCR

A total of 84 isolates of presumptive *Y. enterocolitica* were selected for matrix-assisted laser desorption/ionization time of flight mass spectrometry identification (MALDI-TOF MS, Bruker Daltonik, Bremen, Germany), with detailed description provided in a recent study [18]. 

A total of 65 isolates from this study (representing all positive batches/farms) and 32 isolates from a previous study [16] were selected for Real Time PCR to confirm the presence of the *ail* gene. The number of tested isolates (97 in total) was conditioned by test assays (*n* = 100) provided in the diagnostic kit. The positive control was a human isolate of *Y. enterocolitica* 4/O:3 and the negative controls were two atypical colonies selected from CIN agar and CHROMagar^TM^. DNA isolation was performed using the Gene JET Genomic DNA Purification Kit (Thermo Fisher Scientific, Waltham, WA, USA). PCR amplification and detection was performed according to the protocol of VIASURE *Yersinia enterocolitica* Real Time PCR detection kit (Certest Biotec S.L., Zaragoza, Spain). The sample was positive if the threshold cycle (Ct) value was below 40 and the internal control showed an amplification signal. 

#### 2.4.2. Biotyping, Serotyping, and PFGE Typing of Isolates

Isolates from both surveys (this study: *n* = 84, previous study: *n* = 49) were biotyped according to the standard HRN EN ISO 10273: 2017 [19] using the reactions of esculin, xylose, pyrazinamidase, tween esterase/lipase, trehalose, and indole. Xylose and trehalose solutions, slant agar pyrazinamidase, and Tween esterase/lipase plates were purchased from the Croatian Veterinary Institute, Zagreb. Esculin and indole reactions were tested on Rapid 20E and API 20E, respectively (bioMérieux, Marcy l’Etoile, France). Serotyping was performed by agglutination of *Y. enterocolitica* O:3 antiserum (Statens Serum Institute, Copenhagen, Denmark). Human isolate *Y. enterocolitica* 4/O:3 was used as a positive control (courtesy of Višnja Kružičević, MD, Croatian Institute of Public Health).

Molecular profiles of isolates were compared by PFGE in order to evaluate the possible persistence of specific genotypes in pig tonsils. The PulseNet One-Day (24–28 h) Standardized Laboratory Protocol for Molecular Subtyping of *Yersinia pestis* was used [20]. One rare-cutting restriction enzyme, AscI (New England Biolabs, Beverly, MA, USA) was used for restriction endonuclease digestion. The gels were stained with ethidium bromide and visualized and digitally photographed with a Molecular imager GelDoc XR+ camera system (Bio-Rad Laboratories, Hercules, CA, USA). Fragment size was determined with a low-range CHEF DNA Size Standard Lambda Ladder marker (Bio-Rad Laboratories, Hercules, CA, USA). The PFGE typing results were analyzed with FPQuest software version 5.10 (Bio-Rad Laboratories, Hercules, CA, USA). Dice coefficient with optimization and tolerance set at 1% was used to identify similarities between the PFGE types. A dendrogram was constructed with the unweighted pair group method using arithmetic means showing genetic similarity (percent). The position tolerance was set to 1.5%, with the average optimization value at 1.0%. A down limit for band interpretation at 33kbp was used as recommended for *Salmonella* by Peters et al. [21].

#### 2.4.3. Testing the Susceptibility of *Y. enterocolitica* to Antimicrobial Agents

All isolates (this study: *n* = 84, previous study: *n* = 49) were tested for susceptibility to antimicrobial agents by the disk diffusion method. A 0.5 McFarland cell solution (Densimat, bioMérieux, Marcy l’Etoile, France) was prepared prior to the application of the test isolate on Mueller-Hinton agar (Bio-Rad Laboratories). Eleven antibiotics (MASTDISKS^®^ AST, Mast Group, Bootle, UK) were used: Levofloxacin (5 μg), Ciprofloxacin (5 μg), Ampicillin (10 μg), Cephalothin (30 μg), Cefotaxime (30 μg), Tetracycline (30 μg), Nalidixic acid (30 μg), Ceftazidime 30 μg), Trimethoprim/Sulfamethoxazole (25 μg), Chloramphenicol (30 μg), and Streptomycin (10 μg). Zones of inhibitions were measured by automated system Scan 1200 (Interscience, Saint-Nom-la-Bretèche, France) and interpreted according to CLSI criteria for *Enterobacteriaceae* [22].

### 2.5. Statistical Analysis

In data processing, descriptive statistics methods were used for the quantitative data and data distribution to estimate the curve. Since most of the data were non-parametric, non-parametric tests were used: Spearman’s correlation, Mann—Whitney U test, Kruskal—Wallis test, and Fisher exact test. All data were correlated and tested for differences between slaughterhouses, farms, and years. Depending on the data, the χ^2^ test was used for qualitative data and proportional estimates, the Student’s *t*-test was used to analyze differences between quantitative data between two groups when the data were normally distributed, the Mann—Whitney U test was used for other data distributions, and the Kruskal—Wallis test with multiple rank comparison was used to test multiple groups simultaneously. Differences were significant at the *p* < 0.05 level. The Statistica 13.1 program (Stata Corp., Lakeway Drive, TX, USA) was used.

## 3. Results

### 3.1. Prevalence of Y. enterocolitica in Pig Tonsils and Retail Meat

The study revealed a prevalence of *Y. enterocolitica* in pig tonsils of 43% (Table 2). In pigs from big integrated, medium-sized, and small family farms, the prevalence was 29%, 52%, and 40%, respectively. The percentage of *Yersinia*-positive pigs from integrated farms ranged from 14% to 43%. Although the three integrated farms were in the highest biosecurity category (i.e., category 3), a statistically significant difference in prevalence was found between two of these integrated farms (*p* < 0.05). 

Pigs from medium-sized farms were slaughtered in three slaughterhouses (2, 3, and 4). When *Y. enterocolitica* prevalences were compared depending on the place of slaughter (42%, 86%, and 57% at slaughterhouses 2, 3, and 4, respectively), a significant difference was found between slaughterhouse 2 and slaughterhouse 3 (*p* < 0.05). Considering slaughterhouse 2, the prevalence of positive pigs ranged from 15.4% to 67%, and 39% *Yersinia*-positive pigs originated from medium-sized farms of the highest biosecurity category 3. Comparing this result with the medium farms of lower biosecurity category 2 (58% positive pigs), the difference was not statistically significant (*p* = 0.2104, χ^2^ = 1.568). Similarly, biosecurity category did not significantly affect the proportions of *Yersinia*-positive pigs from medium-sized farms slaughtered in slaughterhouse 3. Excluding the slaughterhouse factor, within pigs from medium-sized farms, 44% of *Yersinia*-positive pigs originated from the highest biosecurity farms, while 60% were from lower biosecurity farms. However, this difference was not significant (*p* = 0.2482; χ^2^ = 1.333). In addition, within biosecurity category 3, no statistically significant differences in *Yersinia* prevalences were found between medium-sized farms and big integrated farms. The majority of family farms (77%) were in lower biosecurity category 2, and 48% (*n* = 29) of the pigs from these farms were *Yersinia*-positive. Compared to the family farms in category 3, the difference was significant (*p* = 0.0460, χ^2^ = 1.333). All retail samples of portioned and minced pork were negative for pathogenic *Y. enterocolitica*. 

### 3.2. Recovery Rates of Y. enterocolitica with Different Isolation Procedures 

As presented in Table 3, the lowest number of positive samples (*Y. enterocolitica* isolated from pig tonsils) was detected when only PSB broth was used followed by streaking on selective agars. The type of agar (CIN or CHROMagar^TM^) did not significantly affect the success of bacterial isolation (*p* = 0.288). Alkali treatment of PSB broth cultures significantly increased the frequency of isolation of pathogenic *Y. enterocolitica*, by 5.4-fold on CIN agar and 3.7-fold on CHROMagar^TM^, respectively (*p* = 0.000; *p* = 0.022) (Table 4). The frequency of *Y. enterocolitica* isolation after alkali treatment of PSB broth cultures was not statistically different with respect to the selective agar used (*p* = 0.05). Compared to PSB broth, enrichment in ITC broth showed a significantly higher number of *Yersinia*-positive tonsils after inoculation on CIN agar or CHROMagar^TM^ (*p* < 0.05). There were no differences in pathogen growth on the selective agars used (*p* = 0.70). KOH treatment of ITC broth cultures also showed an increase in the number of *Yersinia*-positive tonsils detected using CIN agar, but without statistical significance compared to untreated ITC broth (*p* = 0.422). Similarly, the frequency of pathogen isolation on CHROMagar^TM^ was not altered by alkali treatment of ITC broth (*p* > 0.05). Thus, a significantly higher frequency of *Y. enterocolitica* isolation was observed on CIN agar than on CHROMagar^TM^ after alkali treatment of ITC broth cultures (*p* = 0.0002). 

### 3.3. MALDI-TOF MS and Real Time PCR Identification, Bio-, Sero-, and PFGE-Typing 

**Figure 1:**
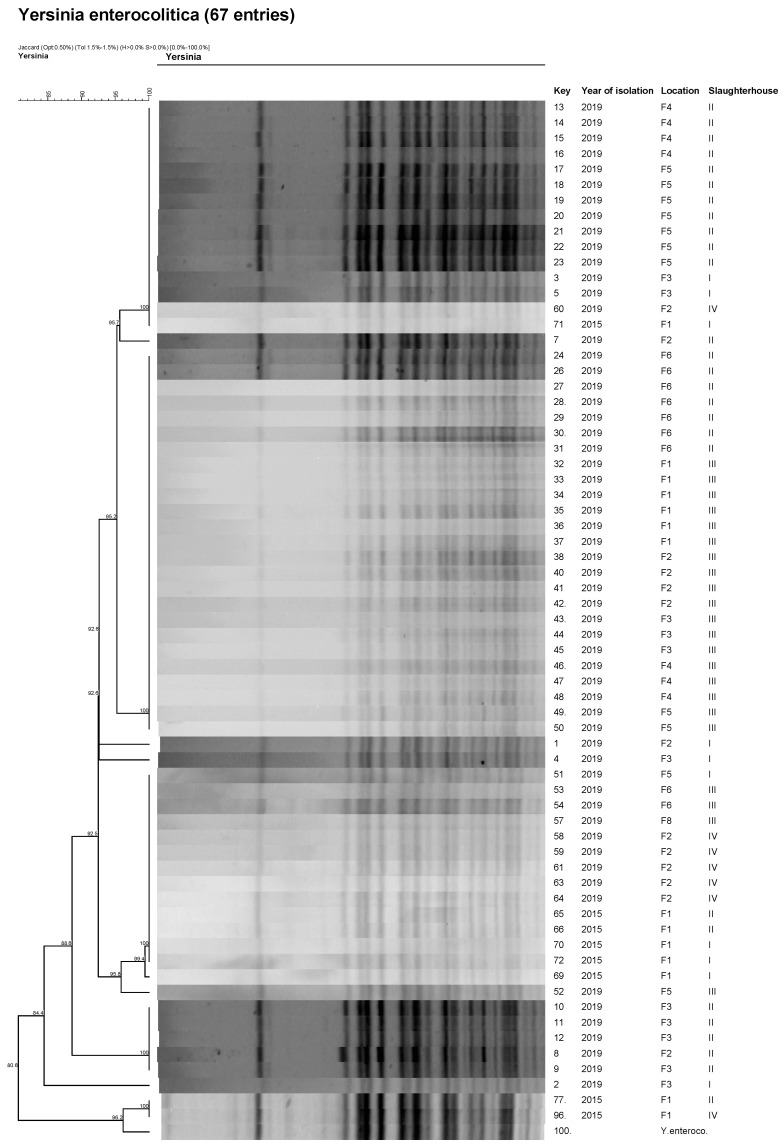
PFGE profiles of *Y. enterocolitica* 4/O:3 from different farm types, slaughterhouses, and years of isolation.

Isolates (*n* = 84) were confirmed by MALDI-TOF MS with a very high probability (score 2.30–3.00) to be *Y. enterocolitica*, while atypical colonies were assigned to *Citrobacter* or *Serratia* species. All the isolates belonged to biotype 4, characterized by negative reactions of aesculin, xylose, pyrazinamidase, lipase, and indole, with a positive reaction of trehalose. Serotyping confirmed that all biotype 4 isolates belonged to serotype O:3, regardless of the year of isolation and the origin of the pigs, i.e., the type of fattening farm. All tested isolates were also positive for the *ail* gene by Real Time PCR. PFGE analysis showed low variability of pulse types within successfully typed (*n* = 66) pathogenic *Y. enterocolitica* 4/O:3 isolates (Figure 1).

### 3.4. Susceptibility of Y. enterocolitica 4/O:3 Isolates to Antimicrobial Agents

In total (both surveys), 36 isolates of *Y. enterocolitica* 4/O:3 from big integrated farms, 84 isolates from medium-sized farms, and 13 isolates from small family farms were tested for susceptibility to 11 antimicrobial agents. Considering isolates from the previous survey (*n* = 49; integrated and medium farms), in addition to natural resistance to ampicillin (92% of isolates) and cephalothin (85%), resistance to chloramphenicol (31%), nalidixic acid (31%), streptomycin (27%), tetracycline (8%), and trimethoprim/sulfamethoxazole (2%) was observed. Only one isolate was sensitive to all antibiotics tested. Among *Y. enterocolitica* 4/O:3 isolates from medium-sized farms only one isolate showed multiresistance (nalidixic acid-chloramphenicol-cefotaxime). In contrast, isolates from big integrated farms were frequently resistant to chloramphenicol, nalidixic acid, and streptomycin. In total, 15 isolates of 24 tested from big integrated farms were multiresistant (Table 5). 

In this study, among the 84 isolates tested, resistance was detected, in addition to ampicillin and cephalothin, toward nalidixic acid (20% of isolates), streptomycin (18%), chloramphenicol (12%), ceftazidime (4.7%), levofloxacin (2.4%), and cephalotaxime (1.2%). Multiresistance was found in 10 isolates among 12 tested from big integrated farms. Nine of these isolates (75%) were simultaneously resistant to nalidixic acid, chloramphenicol, and streptomycin. One isolate was additionally resistant to cefotaxime. In contrast, only one isolate from a medium-sized farm was multiresistant (ceftazidime, trimethoprim/sulfamethoxazole, streptomycin). Similarly, among *Y. enterocolitica* 4/O:3 isolates from family farms, only one multiresistant isolate was found (Table 6). 

Excluding the year of isolation, isolates of *Y. enterocolitica* 4/O:3 from integrated farms were more resistant to streptomycin, chloramphenicol, and nalidixic acid compared to isolates from the other two farm systems (Table 7). No significant differences were found with respect to the susceptibility/resistance of *Y. enterocolitica* isolates from big integrated farms and considering the year of isolation of the pathogen (*p* > 0.05). Similarly, no significant differences were found in the susceptibility/resistance of *Y. enterocolitica* isolates from medium-sized farms between both surveys (*p* > 0.05) (Table 8 and Table 9).

## 4. Discussion

The study was based on the assumption that the overall prevalence of pathogenic *Y. enterocolitica* in the tonsils of pigs does not change significantly depending on the year, but that there are differences related to the type of husbandry, especially in the prevalence of resistant isolates. When pathogenic *Y. enterocolitica* is found in portioned and minced pork, the phenotypic and genetic characteristics of the isolates are expected to be identical to those obtained from the tonsils of pigs from the same farm/slaughterhouse.

### 4.1. Prevalence of Y. enterocolitica in Pig Tonsils at Slaughter and Retail Pork

Given the current lack of data on the prevalence of pathogenic *Y. enterocolitica* in pigs and pork in Croatia, this study aimed to map the production chain from farms to slaughterhouses and pork retail outlets to assess the risk of pathogen transmission to consumers. The relevance of the study stems from the fact that *Y. enterocolitica* is a priority biological hazard in pig meat inspection in Europe and a target of a new comprehensive meat safety assurance system [8,23]. This study builds on the preliminary results previously obtained from a smaller study conducted in 2014, which showed a *Y. enterocolitica* O:3 prevalence of 33% and 10% in tonsils and mandibular lymph nodes, respectively [16]. In comparison, the results of this study showed a higher prevalence of *Y. enterocolitica* in pig tonsils, i.e., 43% [95% CI 36.7–49.7]. The present results are in agreement with other European studies, such as Fredriksson-Ahomaa et al. [24] in Switzerland (prevalence of 34%), van Damme et al. [25] in Belgium (37%), and Martínez et al. [26] in Belgium (44%) and Italy (32%). On the other hand, Fredriksson-Ahomaa et al. [27] and Martínez et al. [26] warned of a high prevalence of pathogenic *Y. enterocolitica* in slaughtered pigs in Finland (62%) and Spain (93%), respectively. At the other extreme are the studies that found low prevalence: 2%, 4%, 8%, 9%, 11%, and 13% [28,29,30,31,32,33]. Several other studies conducted in Europe in recent years also show very different results and the prevalence of *Y. enterocolitica* ranges from 3% [34] (Sardinia), to 14% [35] (Central Italy), to 97% [36] (Finland).

When considering the relationship between *Y. enterocolitica* findings and biosecurity conditions, this study found that there were statistically significant differences in prevalence among integrated farms as well as among medium-sized farms, despite the same level of biosecurity. It is likely that prevalence was affected by slaughterhouse factors, such as possible contact between pig batches at lairage, or omitting sterilization of knife after pluck set removal, as reported before [16].

The opposite was true for family farms, where differences in prevalences were likely related to farm biosecurity levels. Pig farming systems vary among European countries, and comparisons of the prevalence of *Y. enterocolitica* as a function of the type of fattening pig farming system are rare in the literature. However, conventional and alternative (organic) housing systems have been compared, and Nowak et al. [37] found a higher number of positive pigs (29% vs. 18%) in conventional housing systems, with twice as many tonsils from conventionally housed pigs being positive for *Y. enterocolitica* (22% vs. 11%). Also of interest are the results of Novoslavskij et al. [38] in Lithuania, who linked the higher prevalence of *Y. enterocolitica* in pigs to lower farm biosecurity. However, detailed biosecurity factors used in farm categorization were not available in our study, which prevents us from correlating specific factors with observed prevalence. 

In addition, practices at the harvest stage, such as lairage cross-contamination or removal of the pluck set, could influence the rate of contamination of tonsils with *Y. enterocolitica* [39]. All of this highlights the complexity of reporting the true prevalence (pre-harvest) of pathogenic *Y. enterocolitica* and the role of on-farm and slaughter practices in the spread of the pathogen to the consumer. In this context, the assessment of the prevalence of *Y. enterocolitica* based on tonsils as a predilection site needs to be complemented by other data, such as serological tests. In recent years, serological surveillance prior to slaughter has been recommended for risk management purposes in slaughterhouses [40]. Serological testing also showed significant differences in seroprevalence of *Y. enterocolitica* in pigs housed in different fattening systems [41]. Similar to *Salmonella*, data on seroprevalence and/or the presence of *Y. enterocolitica* in lymphoid tissues or intestines can help to reduce risk by implementing decontamination measures on pig carcasses [8].

No positive findings of pathogenic *Y. enterocolitica* were detected when marketed pork cuts and minced pork were examined (*n* = 128), indicating a low risk of *Y. enterocolitica* transmission to such meat. The same results were found in the study by Laukkanen-Ninios et al. [11]. Martins et al. [12] similarly isolated *Y. enterocolitica* from the tonsils and lymph nodes of pigs, but not from environmental samples or from pork cuts. Given slaughter techniques and possible hygiene deficiencies during processing, it is likely that contamination occurs first in the meat of the neck region, head, tongue, and throat, rather than on the carcass, as reported in other studies [11,42]. In contrast to our results, considerable contamination of minced meat with *Y. enterocolitica* was found in other studies [43,44,45,46,47].

#### Recovery Rate of *Y. enterocolitica* by Different Methods of Isolation and MALDI-TOF MS Determination

Another factor that may influence the outcome of determining the prevalence of *Y. enterocolitica* in pig tonsils is the methodology of sampling and isolation. The results obtained show that the success of isolating pathogenic *Y. enterocolitica* by enrichment of tonsils in selective ITC broth is higher than in PSB, but is vice versa after alkali treatment of PSB and ITC broths. Van Damme et al. [25] found that KOH treatment of broth, particularly PSB, was a key factor significantly affecting the success of isolating pathogenic *Y. enterocolitica* from pig tonsils. In our study, we also found that alkali treatment of PSB broth and inoculation on CIN resulted in a significantly higher number of positive samples compared to untreated samples (OR = 7.41, *p* < 0.0001). The same case was found with KOH treatment of PSB and inoculation on CHROMagar^TM^ (OR = 4.71, *p* < 0.0001).

MALDI-TOF MS identification of presumptive colonies demonstrated excellent selectivity of the agars used, especially in the case of CHROMagar^TM^ for screening pathogenic biotypes. This shortens the process for preliminary assessment of pathogenicity, which was determined at later stages by biotyping, serotyping, and detection of the *ail* gene. The use of other chromogenic media, such as YECA, has also been shown to be useful in shortening the process by direct detection of pathogenic biotypes in pig tonsil [48]. In addition, the combination of CHROMagar^®^ and MALDI-TOF MS is less time consuming for the detection of pathogenic isolates compared to conventional isolation methods and biochemical tests. Moreover, MALDI-TOF MS can identify strains belonging to different *Y. enterocolitica* biotypes [49,50]. It is well known that isolation and identification of this bacterium is challenging. Therefore, more sensitive and rapid techniques than existing culture methods have been developed in recent years [9]. Peruzy et al. [51] generally believed that conventional isolation methods for *Y. enterocolitica* are not reliable enough, which they interpreted as due to competition with the background microbiota in tonsils.

### 4.2. Y. enterocolitica Biotyping, Serotyping, PCR, and PFGE Typing

The results obtained from both surveys show the persistence of the pathogenic bioserotype 4/O:3 in the tonsils of fattening pigs in Croatia. This pathogenic bioserotype is most commonly isolated from clinical cases of yersiniosis in humans as well as from carrier pigs in many European countries [52,53,54,55,56]. All *Y. enterocolitica* 4/O:3 isolates from this study carried the *ail* gene that is required for bacterial adhesion and invasion into the host cell as well as serum resistance. However, the gene is also sporadically present in nonpathogenic *Yersinia* species as well as in nonpathogenic *Y. enterocolitica* biotypes such as biotype 1A, so other tests are also needed to confirm the pathogenicity of *Y. enterocolitica* isolates [57]. Therefore, in our study, potential pathogenicity was assessed by colony morphology on chromogenic agar, detection of the *ail* gene, biotyping, and serotyping. The pathogenic bioserotype 4/O:3 is also the prevalent type among *Y. enterocolitica* isolates from fattening pigs sampled at the slaughter line (tonsils) in other European countries, such as Germany (99% of isolates, 2001, [58]), Switzerland (96% of isolates, 2007, [24]), or Finland, 2000, (100%, [3]). The persistence of this bioserotype of *Y. enterocolitica* has been confirmed in similar studies in later years in the same countries [36,59], which is in agreement with our results. In contrast, Bonardi et al. [40,60] reported lower prevalences (15% and 27%) of *Y. enterocolitica* 4/O:3 in two surveys conducted in Italy (2014, 2016). The persistence of the pathogenic *Y. enterocolitica* bioserotype 4/O:3 was recently confirmed in the Brazilian pork production chain (tonsils, oral cavity, head meat) by comparing the results of two studies two years apart, confirming the importance of slaughter hygiene and farming practices in the epidemiology of yersiniosis [13].

Persistence and epidemiology of pathogenic *Y. enterocolitica* is also assessed by molecular typing using methods such as PFGE, MVLA, or whole genome sequencing [9,15]. In our study, selected isolates (based on year of isolation and farm of origin) were subjected to restriction enzyme DNA fragment comparison by PFGE. We found the same pulsotypes occurred regardless of the year of isolation and the origin of the isolates, confirming the assumption of persistence of the pathogenic bioserotype 4/O:3 in pig tonsils. Although the analysis formed several clusters in the dendrogram, their agreement ranged from 80.6% to 100%, indicating low variability of this bioserotype (Figure 1). Similar results from pulsotyping *Y. enterocolitica* bioserotype 4/O:3 isolates were obtained by Martins et al. [13]. They compared pulsotypes of *Y. enterocolitica* bioserotype 4/O:3 isolates collected in 2016 and 2018 from tonsils, lymph nodes, and carcass swabs in the same slaughterhouses using macrorestriction enzymes (XbaI or NotI) and also found high agreement between isolates, ranging from 82.4 to 100%. The low variability of *Y. enterocolitica* 4/O:3 was also noted when comparing human and pig isolates, the pulsotypes of which were combined into a single cluster [61]. Despite the low genetic variability of the 4/O:3 bioserotype, Fredriksson-Ahomaa et al. [62] recommended the PFGE method for distinguishing genotypes present in pig farms using a combination of the restriction enzymes NotI, ApaI, and XhoI. However, the same genotype for bioserotype 4/O:3 isolates was found in most farms (71%). 

### 4.3. Antimicrobial Susceptibility of Y. enterocolitica 4/O:3

In this work, the susceptibility of *Y. enterocolitica* isolates from pig tonsils to antimicrobials was investigated to gain insight into the variability of the resistance profile over time and the origin of the isolates (farm type). The presence of resistant *Y. enterocolitica* in slaughter pigs has been studied in many European countries in recent years [34,40], but not in Croatia. In Latvia [63], resistance to erythromycin and sulfamethoxazole was detected in all *Y. enterocolitica* tested. Bonardi et al. [40], in northern Italy, also reported a frequent prevalence of sulfonamide resistance in slaughtered pigs. In contrast, the prevalence of sulphonamide resistance in our study was rare, as was also reported by other authors from Switzerland and Germany [22,64]. In contrast to other studies [30,65], isolates from the current study were frequently resistant to chloramphenicol, nalidixic acid or streptomycin, and these multiresistant isolates were present in fattening pigs from big integrated farms. In addition, resistance to third generation of cephalosporins was detected in several isolates, which is of clinical relevance. The high public health relevance has been highlighted in recent reports [15] confirming *Y. enterocolitica* 4/O:3 as a novel multidrug-resistant pathogen possessing transmissible resistance determinants.

Therefore, our results show a significantly higher prevalence of multidrug-resistant isolates of *Y. enterocolitica* bioserotype 4/O:3 in big integrated pig farms, although the resistance profile has not changed significantly over the years of research (Table 8). The susceptibility/resistance of *Y. enterocolitica* to certain antimicrobials has also not changed significantly over the years in pigs from medium sized farms (Table 9). To our knowledge, no similar studies have been conducted in Croatia, so more accurate comparisons are not possible. For some bacterial species, resistance profiles can generally be observed with respect to the year of isolation to allow comparison, i.e., insight into an increase or decrease in resistance over time. An earlier study [66] (2007; Switzerland) found that isolates of *Y. enterocolitica* from pork, humans, and pig feces were highly resistant to ampicillin, cephalothin, and amoxicillin/clavulanic acid. In the same year, Fredriksson-Ahomaa et al. [24] found dominant resistance to ampicillin and erythromycin. Bonardi et al. [33] recorded the *Y. enterocolitica* were resistant primarily to cephalothin, ampicillin, streptomycin, and then amoxicillin/clavulanic acid in Italian pig slaughterhouses (2013), and Sacchini et al. [35] reported resistance to ampicillin, streptomycin, sulfisoxazole, tetracycline, nalidixic acid, and chloramphenicol (2018). The resistance profiles of Y. *enterocolitica* have not changed significantly in recent years, likely due to the genetic stability of the pathogen [14]. Fredriksson-Ahomaa et al. [67] found no association between *Y. enterocolitica* genotypes and resistance profiles in pigs. In this context, although our *Y. enterocolitica* 4/O:3 isolates were all genetically similar by the methods used, isolates from the different housing systems showed significant variability in phenotypic antibiotic resistance. This likely reflects the greater exposure of the pathogens to antimicrobial agents on big integrated farms than on small farms.

## 5. Conclusions

Considering all the results presented in this work, the high prevalence of pathogenic *Y. enterocolitica* 4/O:3 in pig tonsils is an important risk factor for pig carcass contamination at slaughter. The pathogen was not isolated from pork cuts or minced meat placed on the market, likely due to good hygiene procedures in meat cutting and preparation, which indicates a low risk to consumers. The low recovery of pathogen from minced meat or pork cuts can also be affected by background microbiota and low sensitivity of culture method. The prevalence of the pathogen in pig tonsils did not depend on the biosecurity level of the farms, except in the case of family farms. Comparison of genetic profiles showed a high concordance of *Y. enterocolitica* isolates over the study years and in the investigated farm systems; the antimicrobial resistance patterns also did not change significantly by year or farm system. However, a significantly higher prevalence of multidrug-resistant isolates was found in pigs from big integrated farms, which could be due to greater pressure of antimicrobial agents used on such farms. 

Further studies of this foodborne pathogen in the context of microbiological safety in pork production chain are needed to gain better insight into antimicrobial resistance and *Yersinia* epidemiology. In addition to culture methods, molecular and serological tests should be used to determine prevalence and distinguish natural infection or transmission from possible external contamination during carcass processing. 

## Figures and Tables

**Table 1:** Characteristics of slaughterhouses included in this study.

Parameter	Slaughterhouse 1	Slaughterhouse 2	Slaughterhouse 3	Slaughterhouse 4
Number of slaughtered fattening pigs per year	308,000	174,000	4000	55,000
Number of slaughtered pigs/h	130	160	20	140
Risk category	High risk	High risk	Medium risk	High risk
Biosecurity of farms (sampled in this study)	3	2–3	1–3	2
Contact between pigs from different farms, lairage	No	Yes *	Yes *	Yes *
Scalding technology	Water (5 min/62 °C)	Steam (20 min/60 °C)	Water (10 min/62 °C)	Water (7 min, 61.5 °C)
Pluck set organ removal techniques and organ placement	Knife, conveyor belt	Knife, hanging hook	Knife, hanging hook	Knife, hanging hook
Head removal and processing on separate line	No	No	No	No

* The pens in the lairage are separated by a fence that allows contact between the pigs.

**Table 2:** Prevalence of *Y. enterocolitica* in tonsils of pigs from different housing systems and slaughterhouses.

Slaughterhouse	Farm Type	Biosecurity	No. of Farms	YE + Farms	No. of Pigs	YE + Pigs (*n*)	YE + Pigs (%)
1	Big integrated	3	3	3	69	20	29%
2	Medium-sized	2 and 3	6	6	74	31	42%
		3	5	5	62	24	39%
		2	1	1	12	7	58%
3	Medium-sized	2 and 3	3	3	14	12	86%
		3	2	2	10	8	80%
		2	1	1	4	4	100%
	Small family farms	1, 2 and 3	13	8	35	14	40%
		3	2	0	5	0	0
		2	10	8	29	14	48%
		1	1	0	1	0	0
4	Medium-sized	3	1	1	42	24	57%
			26	21	234	101	43%

**Table 3:** Comparison of different methods regarding recovery rate and isolation of *Y. enterocolitica* from pig tonsils.

Method of Isolation (Broth + Agar)	Number of Positives (%); *n* = 234	*Y. enterocolitica* Recovery Rate (%); *n* = 101
PSB and CIN	14 (5.9)	13.9
PSB and CHROMagar^TM^	18 (7.7)	17.8
PSB + KOH and CIN	75 (32.0)	74.3
PSB + KOH and CHROMagar^TM^	66 (28.2)	65.3
ITC and CIN	50 (21.4)	49.5
ITC and CHROMagar^TM^	43 (18.4)	42.6
ITC + KOH and CIN	58 (24.8)	57.4
ITC + KOH and CHROMagar^TM^	42 (17.9)	41.6

**Table 4:** *Y. enterocolitica* odds ratio and prevalence ratio between alkali-treated and untreated broths.

Broth and Agar Combinations	Prevalence Ratio	Odds Ratio (OR)	Fisher Exact Test; *p*	Confidence Interval (95% CI)
PSB + KOH and CIN vs. PSB and CIN	5.42	7.41	<0.0001	4.07–13.47
PSB + KOH and CHROMagar^TM^ vs. PSB and CHROMagar^TM^	3.66	4.71	<0.0001	2.71–8.19
ITC + KOH and CIN vs. ITC and CIN	1.15	1.21	0.44	0.78–1.86
ITC + KOH and CHROMagar^TM^ vs. ITC and CHROMagar^TM^	0.97	0.97	1	0.60–1.55

**Table 5:** Prevalence and resistance patterns of multiresistant *Y. enterocolitica* 4/O:3 in pig tonsils from different farm types (2014).

Farm Type	Resistance Pattern	Number of Resistant Isolates	Number of Tested Isolates	% of Multiresistant Isolates/Patterns
Big integrated	NA-CHL-STR	13	24	54
	TET-NA-CHL-STR	1		4
	TET-NA-CAZ-TMP/SMX	1		4
Medium-sized	NA-CHL-CFX	1	25	4

NA: nalidixic acid, CHL: Chloramphenicol, STR: Streptomycin, TET: Tetracycline, CAZ: Ceftazidime, TMP/SMX: Trimethoprim/Sulfamethoxazole, CFX: Cefotaxime.

**Table 6:** Prevalence and resistance patterns of multiresistant *Y. enterocolitica* 4/O:3 in pig tonsils from different farm types (2019).

Farm Type	Resistance Pattern	Number of Resistant Isolates	Number of Tested Isolates	% of Multiresistant Isolates/Patterns
Big integrated	NA-CHL-STR	9	12	75
	TET-NA-CHL-CFX	1		8
Medium-sized	CAZ-TMP/SMX-STR	1	59	2
Small	NA-CAZ-TMP/SMX	1	13	8

NA: nalidixic acid, CHL: Chloramphenicol, STR: Streptomycin, TET: Tetracycline, CAZ: Ceftazidime, TMP/SMX: Trimethoprim/Sulfamethoxazole, CFX: Cefotaxime.

**Table 7:** Antimicrobial susceptibility of *Y. enterocolitica* 4/O:3 isolates collected in two surveys of tonsils from pigs raised in different housing systems.

Antimicrobial Agent	Big Integrated Farms (*n* = 36)			Medium-Sized Farms (*n* = 84)			Small Family Farms (*n* = 13)			Total (*n* = 133)		
	S	I	R	S	I	R	S	I	R	S	I	R
Levofloxacin	36	0	0	81	3	0	13	0	0	130	3	0
Ciprofloxacin	36	0	0	84	0	0	13	0	0	133	0	0
Ampicillin	1	7	28	2	10	72	0	0	13	3	17	113
Cephalothin	2	5	29	16	2	66	0	0	13	18	7	108
Cefotaxime	35	0	1	81	2	1	13	0	0	129	2	2
Tetracycline	34	0	2	83	0	1	13	0	0	130	0	3
Nalidixic acid	9	0	27	76	3	5	12	0	1	97	3	33
Ceftazidime	35	0	1	79	2	3	12	0	1	126	2	5
Trimethoprim/ Sulfamethoxazole	34	1	1	83	0	1	12	0	1	129	1	3
Chloramphenicol	12	0	24	82	1	1	13	0	0	107	1	25
Streptomycin	11	3	22	72	8	4	10	1	2	93	12	28

S = sensitive, I = intermediate, R = resistant.

**Table 8:** Antimicrobial susceptibility/resistance of *Y. enterocolitica* 4/O:3 isolates from big integrated farms.

Antimicrobial Agent	Year 2014 (*n* = 24)			Year 2019 (*n* = 12)		
	S	I	R	S	I	R
Levofloxacin	24	0	0	12	0	0
Ciprofloxacin	24	0	0	12	0	0
Ampicillin	1	2	21	0	5	7
Cephalothin	1	5	18	0	0	12
Cefotaxime	24	0	0	11	0	1
Tetracycline	22	0	2	12	0	0
Nalidixic acid	9	0	15	0	0	12
Ceftazidime	23	0	1	12	0	0
Trimethoprim/Sulfamethoxazole	23	0	1	11	1	0
Chloramphenicol	9	0	15	3	0	9
Streptomycin	9	1	14	2	2	8

S = sensitive, I = intermediate, R = resistant.

**Table 9:** Antimicrobial susceptibility/resistance of *Y. enterocolitica* 4/O:3 isolates from medium-sized farms.

Antimicrobial Agent	Year 2014 (*n* = 25)			Year 2019 (*n* = 59)		
	S	I	R	S	I	R
Levofloxacin	25	0	0	56	0	3
Ciprofloxacin	25	0	0	59	0	0
Ampicillin	2	6	17	0	5	54
Cephalothin	0	2	23	0	0	59
Cefotaxime	22	2	1	59	0	0
Tetracycline	24	0	1	59	0	0
Nalidixic acid	23	0	2	53	3	3
Ceftazidime	24	1	0	55	1	3
Trimethoprim/Sulfamethoxazole	25	0	0	58	0	1
Chloramphenicol	24	0	1	58	1	0
Streptomycin	25	0	0	47	8	4

S = sensitive, I = intermediate, R = resistant.

## Data Availability

Data is contained within the article or supplementary material.

## Supplementary Materials

The following supporting information can be downloaded at: https://www.mdpi.com/article/10.3390/foods11101459/s1, Table S1. Tonsil sampling scheme in slaughtered fattening pigs from different farm types.
