# Supplementary material for: Prevalence and Persistence of Multidrug-Resistant Yersinia enterocolitica 4/O:3 in Tonsils of Slaughter Pigs from Different Housing Systems in Croatia
Source: Foods. 2022 May 18;11(10):1459. doi: 10.3390/foods11101459 (PMC9140555; doi:10.3390/foods11101459)
Supplement: Supplementary file 1 [file foods-11-01459-s001.zip › foods-1642512-supplementary.pdf]

Table S1. Tonsil sampling scheme in slaughtered fattening pigs from different farm types.

Slaughterhouse	Farm type	Farm biosecurity category	Number of sampled pigs (tonsils)	Number of sampled retail meat
1	Big integrated	3	20	
	Big integrated	3	21	12
	Big integrated	3	28	
2	Medium-sized	3	13	
	Medium-sized	3	13	
	Medium-sized	3	12	30
	Medium-sized	3	12	
	Medium-sized	3	12	
	Medium-sized	2	12	
3	Medium-sized	3	7	
	Small family farm	2	2	
	Small family farm	2	2	
	Small family farm	2	2	10
	Small family farm	2	3	
	Small family farm	2	3	
	Small family farm	3	2	
	Medium-sized	3	3	
	Small family farm	2	3	
	Small family farm	3	3	
	Small family farm	2	2	
	Small family farm	1	1	10
	Small family farm	2	2	
	Small family farm	2	5	
	Medium-sized	2	4	
	Small family farm	2	5	
4	Medium-sized*	3	21	30
	Medium-sized*	3	21	

*one single farm
